# A Rare Presentation of Patient With CIDP Found to Have Respiratory Failure and Encephalopathy

**DOI:** 10.1155/crcc/8875501

**Published:** 2025-04-07

**Authors:** Pankti Sheth, Armando Rodriguez Lopez, Shradha Acharya, Bryan Dunn

**Affiliations:** ^1^Brody School of Medicine, East Carolina University, Greenville, North Carolina, USA; ^2^Department of Nephrology and Critical Care Medicine, East Carolina University Health Medical Center, Greenville, North Carolina, USA; ^3^Department of Internal Medicine, East Carolina University Health Medical Center, Greenville, North Carolina, USA; ^4^Department of Pulmonary and Critical Care Medicine, East Carolina University Health Medical Center, Greenville, North Carolina, USA

## Abstract

Chronic inflammatory demyelinating polyneuropathy (CIDP) is a rare neuropathy that presents with progressive weakness, sensory disturbances, areflexia, and ataxia. Respiratory failure and encephalopathy are rare and atypical presentations in patients with CIDP. In this report, we discuss a rare presentation of respiratory failure that required intubation and encephalopathy in a patient presenting with clinical signs of typical CIDP supported by nerve conduction, neuroimaging, and cerebrospinal fluid studies. Treatment with plasma exchange and steroids led to minimal clinical improvement in this scenario. Prompt diagnosis and treatment is important, and further research is warranted to understand associations between CIDP and such rare features.

## 1. Introduction

Chronic inflammatory demyelinating polyneuropathy (CIDP) is an acquired neuropathy that primarily affects the peripheral nervous system. The 2021 European Academy of Neurology/Peripheral Nerve Society (EAN/PNS) guideline describes typical CIDP as having all of the following criteria: progressive or relapsing, symmetric weakness of the proximal and distal muscles of upper and lower limbs, with sensory involvement of at least two limbs which develops over at least 2 months in addition to absent or diminished tendon reflexes in all extremities—sometimes affecting cranial nerves [1]. CIDP variants exist such as focal, multifocal, distal, pure motor, and pure sensory.

The leading theory for pathogenesis of CIDP is cell-mediated and humoral-based immune mechanisms [2]. CIDP is primarily a demyelinating disease with some secondary axonal loss [3].

It is diagnosed using clinical, electrodiagnostic, and other supportive criteria. The electrodiagnostic criteria include abnormalities in motor and sensory conduction suggestive of demyelination. Supportive criteria include clinical improvement after being treated with immunomodulatory agents, ultrasound of median nerve or brachial plexus showing nerve enlargement in at least two sites, magnetic resonance imaging (MRI) showing enhancement or enlargement of nerve roots, cerebrospinal fluid (CSF) with albuminocytological dissociation with elevated CSF protein and leukocyte count < 10/mm^3^, and/or nerve biopsy showing thinly myelinated or demyelinated axons/nodes or perivascular macrophage clusters [1].

We present a patient who met several diagnostic criteria of CIDP who presented with 3 months of progressive weakness in her bilateral lower and upper extremities in addition to numbness and tingling in her hands and feet, severe acute hypoxemic respiratory failure, and acute encephalopathy that required admission to the intensive care unit (ICU). We discuss the rare presentation of this case with supportive evidence and similar presentations through pertinent literature review.

## 2. Case Presentation

A 58-year-old woman with a history of hypothyroidism, noninsulin-dependent Type 2 diabetes mellitus with diabetic polyneuropathy, colon cancer treated with resection and chemotherapy, thyroid cancer treated with thyroidectomy, chronic hepatitis C virus (HCV), latent syphilis, polysubstance use (cocaine, marijuana, and alcohol), and major depressive disorder with psychotic features presented with 3 months of progressive weakness in her bilateral lower limbs which progressed to her upper limbs making her wheelchair bound. She also experienced gait ataxia, numbness and tingling in her hands and feet, and fecal incontinence around the same time. The family found her unresponsive one morning and brought her to the hospital on July 21, 2024. Notably, on presentation, her blood glucose was low at 38 mg/dL which was corrected with dextrose (D50).

Her physical exam was remarkable for absent motor strength, unresponsiveness to painful stimuli, and absent deep tendon reflexes in the bilateral upper and lower extremities. She did not have a corneal, pupillary, gag, or cough reflex. She required endotracheal intubation and mechanical ventilation for airway protection on the day of her admission. Extensive workup was pursued.

Her lumbar puncture showed CSF remarkable for albuminocytologic dissociation (elevated protein of 132 mg/dL and total nucleated cell count of 0/*μ*L), low glucose of 35 mg/dL, and a negative meningitis/encephalitis CSF panel for *Haemophilus influenza*, *Listeria monocytogenes*, *Neisseria meningitidis*, *Streptococcus agalactiae*, *Streptococcus pneumonia*, cytomegalovirus, Herpes Simplex Virus 1 and 2, enterovirus, varicella zoster, and *Cryptococcus neoformans*.

**Figure 1:**
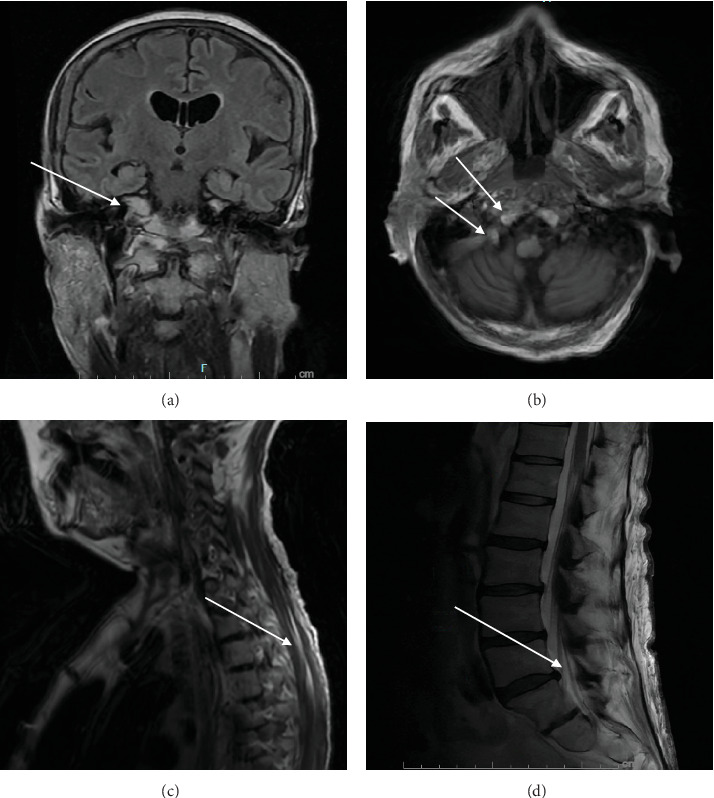
MRI brain: Subtle abnormal enhancement along the trigeminal nerves (a, arrow) and the seventh and eighth cranial nerves (b, arrows) at the internal auditory canal. MRI cervical spine: (c) partially visualized leptomeningeal enhancement of the upper cervical cord. MRI lumbar spine: (d) abnormal leptomeningeal enhancement and enhancing lumbar nerve roots. This is nonspecific and can be seen with infectious and inflammatory conditions including Guillain–Barré syndrome (GBS).

MRI of the brain showed no acute infarction or intracranial abnormality; however, it showed nonspecific, patchy, low attenuation in the periventricular deep white matter typical of chronic microvascular changes. It also displayed subtle abnormal enhancement along the fifth, seventh, and eighth cranial nerves and along the leptomeningeal margin of the upper cervical cord, and chronic right orbital blowout fracture (Figure 1a,b). MRI of the spine showed subtle abnormal enhancement along the upper cervical cord, lumbar nerve roots, and upper cauda equina (Figure 1c,d). Continuous electroencephalogram (EEG) showed continuous slowing and was negative for seizures.

Electromyography (EMG) nerve conduction study found significantly prolonged motor distal latencies, decreased conduction velocities, decreased amplitude, and prolonged F-wave latency difference which suggested demyelination with axonal loss as seen in CIDP (Tables 1 and 2).

Serum motor neuropathy panel was negative for anti-GQ1b, anti-GD1a, anti-GD1b, anti-GM1, and anti-MAG antibodies; however, it demonstrated elevated IgG (2449 mg/dL) and IgA (509 mg/dL).

She had chronic HCV infection with positive HCV antibody. Her viral load was elevated with HCV RNA of 4 million IU when tested during admission (July, 2024) which was higher than previous levels of 2 million IU in June, 2024 and 833,000 IU in 2020. However, she had normal liver function tests and no signs of decompensated cirrhosis. It was unknown if she received treatment for the infection. Her serum cryoglobulin levels were negative with normal complement C4 levels which suggested her peripheral symptoms were not secondary to cryoglobulinemia in the setting of HCV. There have been case reports that show HCV as a potential trigger of CIDP [4, 5] and HCV-related encephalomyelitis [6]. The patient had a history of latent syphilis which was treated with penicillin G and doxycycline. Her fluorescent treponemal antibody absorption (FTAB) was positive; however, her VDRL was negative with no CSF findings suggestive of neurosyphilis.

The progressive symmetric weakness in the bilateral upper and lower extremities along with areflexia over 3 months, CSF findings of albuminocytologic dissociation, EMG nerve conduction study showing demyelinating neuropathy and axonal loss findings with significantly prolonged motor distal latencies, and MRI findings of leptomeningeal and nerve root enhancement highly support the diagnosis of CIDP.

Patient underwent initial therapy of CIDP with five sessions of plasmapheresis (last session on August 3, 2024). She also received 1 g of intravenous methylprednisolone for 5 days around the same time. While receiving treatment, she showed nonpurposeful movements of the head and left upper and lower extremities. She had a return of cough and gag reflex with an eventual return of corneal and pupillary reflex. She opened both of her eyes and turned her head toward the voice when her name was called and withdrew to painful stimulus elicited on the left upper extremity only. This was a mild improvement compared to her initial presentation. Unfortunately, she did not show any other meaningful clinical improvement and remained intubated during her stay in the ICU.

## 3. Discussion

The diagnosis of CIDP should be considered when a patient presents with progressive, symmetric weakness of the proximal and distal muscles, sensory disturbances, areflexia, and gait ataxia over a course of two or more months as seen in the case presented. AIDP (acute inflammatory demyelinating polyneuropathy) presents with similar clinical features; however, one of the required criteria states that the nadir must be reached within < 4 weeks from symptom onset [7]. In this case, the patient's symptoms kept worsening over the course of 3 months which is not consistent with the diagnosis of AIDP.

Diagnosis of CIDP can be made using clinical, electrodiagnostic, and other supportive criteria that has been aforementioned. The initial therapy for CIDP includes intravenous immune globulin (IVIG), plasma exchange, or glucocorticoids. About 66% of patients with CIDP initially respond to therapy while 10%–15% of patients are resistant to all therapies [8]. In this case report, the patient showed minimal improvement after being treated with five sessions of plasma exchange and steroids.

The specific triggers remain unclear but prior research has shown association of CIDP with hepatitis B or C, human immunodeficiency virus, systemic lupus erythematosus, thyroid disorders, diabetes mellitus, nephrotic syndrome, and inflammatory bowel disease [1, 4, 5, 9].

Although respiratory failure is not a typical clinical feature in CIDP, there are case reports of patients with CIDP who had respiratory failure that required admission to ICU with some requiring intubation [10]. Another case report explored four patients with CIDP who had respiratory failure secondary to diaphragmatic paralysis in the setting of phrenic nerve palsy with two requiring ventilatory support [11]. Of note, not all patients responded to treatments. Studies have shown that patients with CIDP who develop respiratory failure tend to have phrenic nerve involvement and diaphragmatic paralysis [11, 12]. Phrenic nerve conduction studies have been shown to have increased response latencies, EMG can confirm loss of motor units in diaphragm, and postmortem findings have shown axonal loss in the phrenic nerve [12–14]. The patient in this case was not subjected to phrenic nerve conductions studies or EMG of the diaphragm.

Central nervous system (CNS) involvement in CIDP has been reported in a few prior studies [15–17]. HCV-related acute encephalopathy could also have contributed to the patient's altered consciousness as well as initial low glucose and oxygen levels [6]. However, hypoglycemic encephalopathy and hypoxic encephalopathy usually present with focal or diffuse lesions on MRI of the brain [18, 19] which were not found in this case presentation.

Given her respiratory failure requiring mechanical ventilation and encephalopathy, Bickerstaff's brainstem encephalitis (BBE), a Miller–Fisher variant of Guillain–Barré syndrome was considered; however, the patient's presentation with areflexia and absent anti-GQ1B antibodies argued against that diagnosis [20]. MRI findings of high-intensity areas on T_2_-weighted images of the brainstem, thalamus, cerebellum, and cerebrum may be seen in BBE [20] whereas patients with Miller–Fisher syndrome may present with enhancement of oculomotor, abducens, and facial nerves bilaterally with no brainstem abnormalities on the MRI [21]. These imaging findings were not appreciated in the MRI scans of the patient presented in this case report further making BBE and Miller–Fisher as the less likely diagnoses.

The patient meets the clinical, electrodiagnostic, and supportive (CSF and MRI findings) criteria based on the 2021 EAN/PNS guidelines which strongly supports the diagnosis of CIDP [1]. CIDP primarily affects the peripheral nervous system; however, few studies have shown concomitant presence of respiratory failure and CNS involvement. In this case presentation, the patient has multiple comorbidities. We state the potential etiologies of the respiratory failure and encephalopathy and explain the reasoning behind the most and least likely etiologies. CIDP develops over at least 8 weeks as seen in this patient. She eventually develops respiratory failure and encephalopathy. We provide evidence that helps exclude other pathologies that could be responsible for such atypical symptoms. Further investigation is warranted to explore cases of CIDP with this atypical involvement and examine treatment options, response, and prognosis.

## 4. Conclusion

Progressive weakness of proximal and distal muscles, sensory involvement, areflexia, and gait ataxia over a course of eight or more weeks should prompt investigation of CIDP. The diagnosis can be supported by characteristic EMG nerve conduction findings, neuroimaging, lumbar puncture findings, and nerve biopsy. In rare cases, patients with CIDP are found to have concomitant presence of respiratory failure and CNS involvement. Early recognition and management are important while further investigation is needed to identify prevalence as well as efficacious therapies for such rare variants.

## Figures and Tables

**Table 1:** Nerve conduction study of motor nerves.

**Stimulation site**	**Onset (ms)**	**Normal onset (ms)**	**O-P Amp (mV)**	**Normal O-P amp**	**Amp (%)**	**Negative area (mVms)**	**Site 1**	**Site 2**	**Dist (cm)**	**Vel (m/s)**	**Normal Vel (m/s)**
*Left fibular motor (extensor digitorum brevis)*											
Ankle	⁣^∗^**9.1**	< 6.1	⁣^∗^**0.8**	> 2.0	100	5.80	Below fibular	Ankle	33	⁣^∗^**29**	> 38
Below fibular	20.3		0.5		62.5	3.56	Popliteal	Below fibular	11	⁣^∗^**35**	> 38
Popliteal	23.4		0.9		180	4.73					
*Right fibular motor (extensor digitorum brevis)*											
Ankle	⁣^∗^**13.3**	< 6.1	⁣^∗^**0.7**	> 2.0	100	2.58					
*Left median motor (abductor pollicis brevis)*											
Wrist	⁣^∗^**5.4**	< 4.5	4.0	> 4	100	21.15	Elbow	Wrist	32.5	⁣^∗^**45**	> 49
Elbow	12.7		2.2		55	10.29					
*Left tibial motor (abductor hallucis brevis)*											
Ankle	⁣^∗^**9.1**	< 6.1	⁣^∗^**1.7**	>3.0	100	5.52	Popliteal fossa	Ankle	41	⁣^∗^**28**	> 35
Popliteal fossa	23.9		1.3		76.5	4.31					
*Right tibial motor (abductor hallucis brevis)*											
Ankle	⁣^∗^**8.6**	< 6.1	⁣^∗^**1.2**	> 3.0	100	7.27					
*Left ulnar motor (abductor digiti minimi)*											
Wrist	⁣^∗^**4.9**	< 3.7	⁣^∗^**2.0**	> 5	100	9.83	Below elbow	Wrist	22	⁣^∗^**45**	> 49
Below elbow	9.8		1.9		95	9.08	Above elbow	Below elbow	14	⁣^∗^**30**	> 49
Above elbow	14.4		1.9		100	9.73					

*Note:* O-P Amp, onset-peak amplitude; Amp, Amplitude; Dist, distance; vel, velocity.

∗Abnormal.

**Table 2:** Nerve conduction F-wave studies.

	**F-latency (ms)**	**Latency normal (ms)**	**Left-right F-latency (ms)**	**Left-right latency normal**
Left median (abductor pollicis brevis)				
⁣^∗^No response		< 33		< 2.2
Left tibial run #2 (abductor hallucis)				
	52.64	< 61	⁣^∗^**11.49**	< 5.7
Right tibial run #2 (abductor hallucis)				
	41.15	< 61	⁣^∗^**11.49**	< 5.7

∗Abnormal.

## Data Availability

Data sharing is not applicable to this article as no new data were created or analyzed in this study.
